# Wounds of the Lungs in the Present War

**Published:** 1917-09

**Authors:** Edward Archibald

**Affiliations:** Assistant Surgeon, Royal Victoria Hospital, Montreal; Lecturer in Clinical Surgery, McGill University


### WOUNDS OF THE LUNGS IN THE PRESENT WAR.

### A Short Review from the Surgical Standpoint.

By Edward Archibald, M.D.,

**Assistant Surgeon, Royal Victoria Hospital, Montreal; Lecturer in Clinical Surgery, McGill University.**

In the present paper the writer does not desire to do more than present a very summary consideration of the subject indicated in the title. As the war has progressed and the number of cases has increased, and observation has become more close, new problems have unfolded and new work has become obviously necessary and is being done, so that to do justice to the general subject would require much more space than is available.

The clinical experience of the writer comprised, approximately, some two hundred cases in all, of which about sixty were seen at a casualty clearing station some five or six miles behind the front line trenches, while the rest came to observation at a base hospital in France, to which the chest cases were sent usually five days or more after the receipt of their injury, although during rushes they were sent down within two or three days.

The clinical signs and symptoms varied a great deal according as the cases were seen at the front or at the base. At the front they usually arrived in the casualty clearing station within three to twelve hours from the injury, although a few, chiefly those suffering from urgent dyspnea and sever hemorrhage, were sometimes kept one or two days in the field ambulance. The striking symptom in nearly all was dyspnea with orthopnea. The patients suffered on the whole very little from severe shock. Their blood-pressure was almost normal or nearly normal, but the degree of dyspnea was often to the uninitiated quite alarming; particularly was it apt to be so if there were present an open wound in the chest wall, establishing an acute open pneumothorax. Frequently with the movements of respiration blood would pour out in quantities and be sucked back. In such cases relief was most readily got by plugging the wound air-tight, converting the open into a closed pneumothorax, and thus saving the mediastinum. Morphia was the great standby, and its effect was quite remarkable. In some there was noted a most severe and apparently spasmodic pain in the region of the diaphragm, together with intractable cough. These cases were best relieved by heroic doses of morphia. However, and fortunately, neither pain nor cough was a troublesome symptom in the majority of the patients. Their chief distress was from shortness of breath.

On physical examination a hemothorax was found with great constancy, much more so, for instance, than hemoptysis. It is fairly certain that the blood came in most instances from the wound in the lung, but in some it certainly originated in a wound of the intercostal vessel. The amount of blood effused, as shown by percussion, was nearly always sufficient to give dulness up to the angle of the scapula, but frequently it went higher than this, and, rarely, it filled the whole chest.

Fever was constantly present. When infection remained absent is usually did not go above 101 degrees, and came down to normal in four or five days. In some cases moderate fever would last eight, ten, or even fourteen days, without our being able in repeated punctures to find any infection.

The improvement of the patient in the first day or two, after being put to bed, was often most remarkable, particularly in the dyspnea. We learned to regard our chest cases as not being, on the whole, anxious ones; nevertheless, in perhaps three or four per cent. the patient would die, of cardiac as well as respiratory failure, from the accumulation of air and blood in the chest cavity. This happened in two of the sixty-odd cases seen at the front. They were difficult, within the first twelve to eighteen hours, to distinguish from the ordinary dyspneic case which gets better, and in the rush of a large convoy they both slipped past us and died without the obviously necessary operation of a thoracotomy. One of these was certainly a valvular pneumothorax; at post-mortem air under tension was found in one side of the chest. This condition is certainly a rare one and therein contrary to my previous expectations. In fact, pneumothorax in general was remarkable by its absence. It may be, as Prof. C. P. Howard suggests, that small amounts of air are frequently present in the first two or three days, but are unrecognized; but if so, the fact remains that careful physical examination at the front as well as at the base usually failed to yield any signs by which pneumothorax could be established. It is true, as we found at the base, from our work in injecting oxygen following the aspiration of blood, that the textbook signs of air in the chest were not to be got in many of the cases.

Operation in the casualty clearing station was very rarely necessary. The patients were usually kept five days or more for safety, and were then sent down to the base. During these few days infection seemed to be decidedly infrequent. If present, it was apt not to go ahead fast enough to render diagnosis obvious, and most empyemata were operated upon after reaching the base. From letters recently received, better work is now being done in the way of early exploratory punctures and bacteriological investigation, with the result that early empyemata are being found and operated upon more frequently.

Our experience at the base was in many respects quite different from that just described. The patients usually arrived in fairly good condition with little if any dyspnea, largely recovered from the distress of the first few days, and with a hemothorax which had reached its maximum and was stationary. The danger he was in was no longer that of hemorrhage or of mechanical interference with respiration and heart action; what we had to fear was chiefly infection. Accordingly, in such cases as still showed fever, the first thing done was to remove some blood for bacteriological examination. Now the finding of infection was frequently not the simple matter that it might appear to be. While the blood effused into the pleaura nearly always remains fluid, for a long time, there is, nevertheless, in many cases, some degree of clotting, with the result that the pleural space might be divided up into two or more walled off cavities, and the blood removed by one puncture, might be sterile, while that from another puncture, in a different spot, would be infected. It therefore became the rule in the presenct of fever, prolonged more than eight or nine days, or before that lapse of time, when clinical symptoms suggesting infection were present, to repeat punctures in different places until one had assured oneself that blood from all parts of the cavity had been examined. It happened to us on a number of occasion to get a positive becteriological finding on the third, fourth, or fifth punctures only. Another point was that bacteria apparently can be present in clots, and not break out and infect the general mass of blood for some days. The problem was thus often a more complicated one than we ar accustomed to encounter in the ordinary empyemata of civil practise.

It was astonishing to find in how few cases, comparatively, infection did develop. Of course with clean bullet wounds it practically did not occur, but we had expected that fragments of high explosive would very often indeed carry infection. So little was this the case that, speaking generally, empyema occurred in our cases only in about 14 per cent. (11 to 82 analyzed). Of the empyemata which did develop the mortality was on the whole higher than in civil practice. Of the 11 cases just mentioned four died; one from a streptococcus and anaerobic infection; two from a streptococcus septicemia, of which two, one really died from a secondary hemorrhage; and the fourth from the combination of streptococcus infection with multiple injuries. Of those which recovered, seven in number, six had streptococcus infection, one complicated by a streptococcus septicemia, and the seventh was chiefly infected by fecal bacilli.

In treatment the ordinary rib resection was done. While I am strongly in favor of suction drainage with air-tight dressings in the ordinary cases of civil work, I found it frequently impossible in view of the nature of the wounds to apply this during the war, and it did not always prove beneficial even in those casese where it was applied. That form of drainage needs such careful attention to detail, and war condition so frequently do not allow this, that in some cases it was found safer to drain as usual with the ordinary big empyema tube; otherwise retention sometimes occurred.

In those cases that remained sterile our practice was to aspirate practically all of the pleural blood anywhere from the seventh to the twelfth day, but preferably early, and to replace simultaneously, but through a second needle, an amount of oxygen corresponding roughly with the amount of blood removed. This was to prevent secondary oozing. I doubt if it had any such effect, but I continued it because it made the aspiration so easy and painless. The usual distressing cough towards the close of aspiration was always prevented The amount removed was usually from fifteen to thiry ounces, but I have taken off as much as eightly at one sitting without harm. We always did this under control of manometer readings of intrapleural pressure. It is interesting to note that this way very rarely positive, and then only to the extent of 2 to 4cm. (water) at the end of expiration. Negative pressure during inspiration ran usually from 8 to as high as 16 to 18 cm. of water.

One interesting observation I might mention which has a bearing on the question of the displacement of the heart in pneumothoraz. In two of the very few pneumothoraz cases we had, I took the intrapeural pressure on both sides simultaneously. Although the heart in both was greatly displaced, the intrapeural pressure on the side of the pneumothorax was in one case equal to that of the normal opposite pleura, while in the other case it measured only some 2 to 4 cm. of water more than on the normal side.

Just a few words in conclusion concerning the question of the removal of foreign bodies embedded in the lung. The English practice was to leave them alone. Among the French surgeons there grew up during 1915 and 1916 a strong tendency to remove all except the smallest. Marion, Duval, and a Bordeaux surgeon reported series of from 30 to 60 cases with practically no mortality, each upholding a different method. For myself, after the experience of removing some four or five, I came to the conclusion that from no point of view was it justifiable within at least the first month or two to remove foreign bodies by operation unless it could be shown that an abscess was developing around them. Now this is an interesting and somewhat surprising fact, that lung abscess around a foreign body, at any rate during the three to five weeks' stay of the men in France, was a thing of the utmost rarity. Whether it occurred later on in England I was quite unable to ascertain, although I questioned several of the consultants about it. It seems, therefore, that when it has been shown that foreign bodies become encapsulated and are very little liable to go on to infection unless late, it is not our part to risk the dangers of empyema, traumatic pneumonia, and possibly hemorrhage, in the attempt to remove these bodies by early operation.—International Journal of Surgery.

